# Recent Advances in Transition Metal Dichalcogenide Cathode Materials for Aqueous Rechargeable Multivalent Metal-Ion Batteries

**DOI:** 10.3390/nano11061517

**Published:** 2021-06-08

**Authors:** Vo Pham Hoang Huy, Yong Nam Ahn, Jaehyun Hur

**Affiliations:** Department of Chemical and Biological Engineering, Gachon University, Seongnam 13120, Gyeonggi, Korea; vophamhoanghuy@yahoo.com.vn (V.P.H.H.); yahn@gachon.ac.kr (Y.N.A.)

**Keywords:** transition metal dichalcogenide, aqueous multivalent metal-ion batteries, zinc-ion batteries, magnesium-ion batteries, aluminum-ion batteries

## Abstract

The generation of renewable energy is a promising solution to counter the rapid increase in energy consumption. Nevertheless, the availability of renewable resources (e.g., wind, solar, and tidal) is non-continuous and temporary in nature, posing new demands for the production of next-generation large-scale energy storage devices. Because of their low cost, highly abundant raw materials, high safety, and environmental friendliness, aqueous rechargeable multivalent metal-ion batteries (AMMIBs) have recently garnered immense attention. However, several challenges hamper the development of AMMIBs, including their narrow electrochemical stability, poor ion diffusion kinetics, and electrode instability. Transition metal dichalcogenides (TMDs) have been extensively investigated for applications in energy storage devices because of their distinct chemical and physical properties. The wide interlayer distance of layered TMDs is an appealing property for ion diffusion and intercalation. This review focuses on the most recent advances in TMDs as cathode materials for aqueous rechargeable batteries based on multivalent charge carriers (Zn^2+^, Mg^2+^, and Al^3+^). Through this review, the key aspects of TMD materials for high-performance AMMIBs are highlighted. Furthermore, additional suggestions and strategies for the development of improved TMDs are discussed to inspire new research directions.

## 1. Introduction

Nowadays, owing to their depleting sources and adverse effects on the environment, traditional fossil fuels have been replaced by various renewable sources, such as sunlight, tides, and wind. However, the non-continuous supply of these renewable resources has sparked the need to develop safe and low-cost energy storage devices, especially for large-scale grid applications [1,2,3]. Lithium-ion batteries (LIBs) have been extensively investigated as an energy resource solution for grid applications and as the primary source of electrical machines owing to their high energy/power density and durability [4,5,6,7,8,9,10,11,12,13,14,15,16]. Nevertheless, the low availability of lithium, its growing cost, and safety issues hamper the application of these batteries in the production of commercial storage devices. Other monovalent metal-ion batteries, namely sodium-ion batteries (SIBs) and potassium-ion batteries (KIBs) have also been widely researched owing to their similar chemical properties to those of LIBs and the relatively higher abundance of Na and K [17,18,19,20,21]. Nevertheless, like LIBs, SIBs and KIBs use highly toxic electrolytes, which raise safety concerns and increase the fabrication costs.

Compared to batteries using organic electrolytes, aqueous rechargeable batteries (ARBs) possess tremendous competitive potential for energy storage devices because of their outstanding advantages such as: (i) high safety due to the absence of harmful solvents with high volatility; (ii) low cost and environmental friendliness; (iii) high ionic conductivity of aqueous electrolytes compared to that of organic electrolytes (1000 times higher); and (iv) the absence of a solid electrolyte interphase (SEI) layer during the electrochemical reaction [22,23,24]. Therefore, ARBs can potentially meet the demands of high energy density, high elemental abundance, and better safety features [25,26]. In particular, the use of aqueous multivalent metal-ion batteries (AMMIBs) is an ingenious solution for meeting the rapidly increasing demand for high-performance and cost-effective energy storage devices. According to the data in Table 1, multivalent metal anode has high volumetric energy density and theoretical capacity due to the multi-electron transfer capability. Consequently, various efforts have been made to study the various types of AMMIBs, such as zinc-ion batteries (ZIBs) [27,28,29,30], magnesium-ion batteries (MIBs) [31], aluminum-ion batteries (AIBs) [32], and calcium-ion batteries (CIBs) [33].

Transition metal dichalcogenides (TMDs) have attracted significant attention as potential materials in diverse applications pertaining to energy storage [34,35,36,37,38]. TMDs with the general formula MX_2_ (M: transition metal, X: chalcogen) are often considered as inorganic analogues of graphite. Every single TMD layer is formed by three atoms (X-M-X) with an M layer sandwiched by two X atomic layers. The weak interlayer van der Waals (vdW) force facilitates the insertion of ions [39,40,41,42]. This property is especially adequate for AMMIB electrodes that utilize large hydrated metal ions. For example, molybdenum disulfide (MoS_2_) with an interlayer distance of 0.62 nm is considered as a representative member of the TMD family and can easily accommodate hydrated Zn^2+^ ions in its framework. Nevertheless, numerous studies have demonstrated the difficulty of Zn^2+^ storage in bulk MoS_2_ [24]. This indicates the incompatibility of MoS_2_ as a ZIB cathode despite its sufficient interlayer distance. Consequently, the simple adoption of TMDs as potential cathodes is not a great strategy for realizing high-performance ZIBs.

**Table 1:** Comparison of multivalent and monovalent metal-ions [23,30,33,43,44,45,46].

Charge Carrier Ions	Zn^2+^	Mg^2+^	Al^3+^	Ca^2+^	Li^+^	Na^+^	K^+^
Atomic mass (g mol^−1^)	65.41	24.31	26.98	40.08	6.94	22.99	39.1
Density (at 20 °C) (g cm^−3^)	7.11	1.74	2.7	1.55	0.53	0.97	0.89
Crystal structure	Hexagonal	Hexagonal	face-centered cubic	face-centered cubic	body-centered cubic	body-centered cubic	body-centered cubic
Abundance ^a^	25	8	3	5	33	6	7
Metal cost (USD kg^−1^)	2.2	2.2	1.9	2.28	19.2	3.1	13.1
Ionic radius (Å)	0.75	0.72	0.53	1.00	0.76	1.02	1.38
Hydrated ionic radius (Å)	4.3	4.28	4.75	4.12	3.82	3.58	3.31
Redox potential vs. SHE	−0.763	−2.356	−1.676	−2.84	−3.04	−2.713	−2.924
Gravimetric specific capacity of metal anode (mAh g^−1^)	820	2206	2980	1337	3860	1166	685
Volumetric specific capacity (mAh cm^−1^)	5855	3834	8046	2072	2061	1129	610

^a^ Ranking on the basis of the abundance of all the elements on earth.

**Figure 1:**
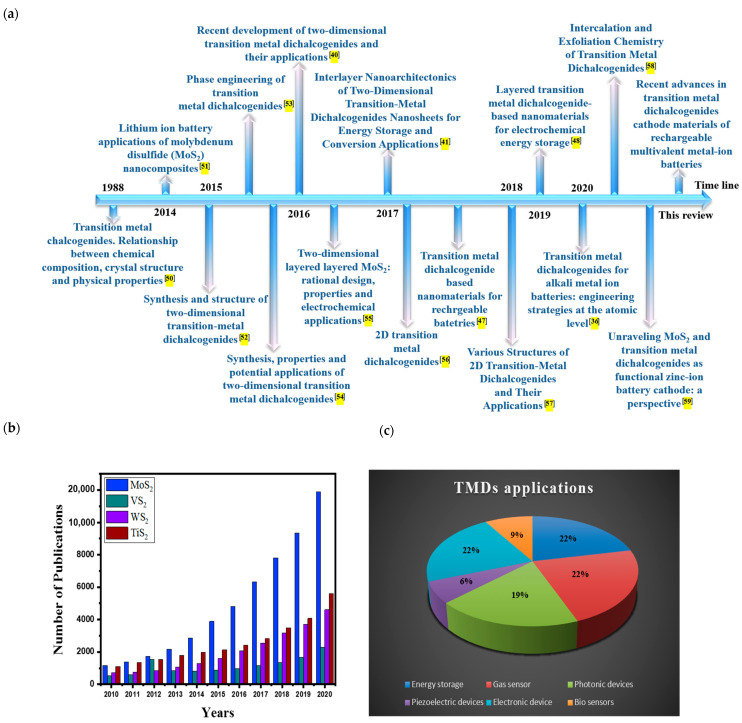
(**a**) Brief summary of recent reviews on transition metal dichalcogenides (TMD) materials, (**b**) Year-wise publication plot for TMD materials including MoS_2_, VS_2_, WS_2_, and TiS_2_ in the period of 2010–2020. (searched by Google Scholar, 2 June 2021), (**c**) The applications of TMDs in the period of 2010–2020.

Recently, there have been many reviews focusing on the TMD materials [47,48] and other cathode materials [49] for battery applications. While most previous reviews on TMD materials for battery applications covered various rechargeable batteries regardless of the electrolyte type [47,48], the reviews for the cathode materials dealt with a wide range of cathode candidate materials beyond TMDs [49]. The main viewpoint in this review is centered on the TMD materials as a cathode and multivalent metal ions dissolved in water as a electrolyte, respectively, which is distinctively different from most previous review articles. Figure 1a,b highlight some recent reviews and the numbers of publications on TMDs over the last 10 years [36,40,41,47,48,50,51,52,53,54,55,56,57,58,59]. The number of publications on various TMD materials has been steadily increased from the year of 2010. In particular, the increase in publication number has been more radical since the year of 2015. In the perspectives of applications, TMDs have been widely used in energy, sensors, optoelectronic devices, piezoelectric devices, and biosensors (Figure 1c). Considering the high interest in energy storage using TMD materials, the reviews focusing on the AMMIB cathode materials have been rare. To stimulate new research strategies in this direction, this review provides an overview of TMDs as cathode materials in AMMIB. This review summarizes the current developments of TMD cathodes in various AMMIBs and discusses the main challenges as well as the advantages of employing TMDs in AMMIBs. Some modification strategies for TMDs have also been discussed to enhance multivalent-ion storage in aqueous batteries. Finally, the future perspective and outlook toward next-generation AMMIB cathode research will be discussed, which would be beneficial for the rational design of TMD-based materials for AMMIBs. 

## 2. Brief Introduction of Transition Metal Dichalcogenides (TMDs)

### 2.1. Concept and Principle of TMDs

**Figure 2:**
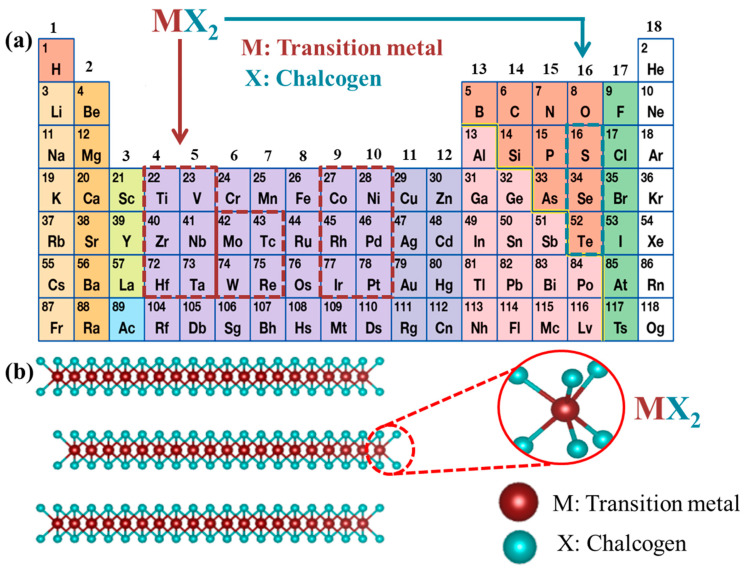
(**a**) TMDs with the MX_2_ structure consisting of M from the 16 transition metals indicated by the red dotted box and X from the three halogen elements indicated by the green dotted box, (**b**) layered structure of MX_2_.

**Figure 3:**
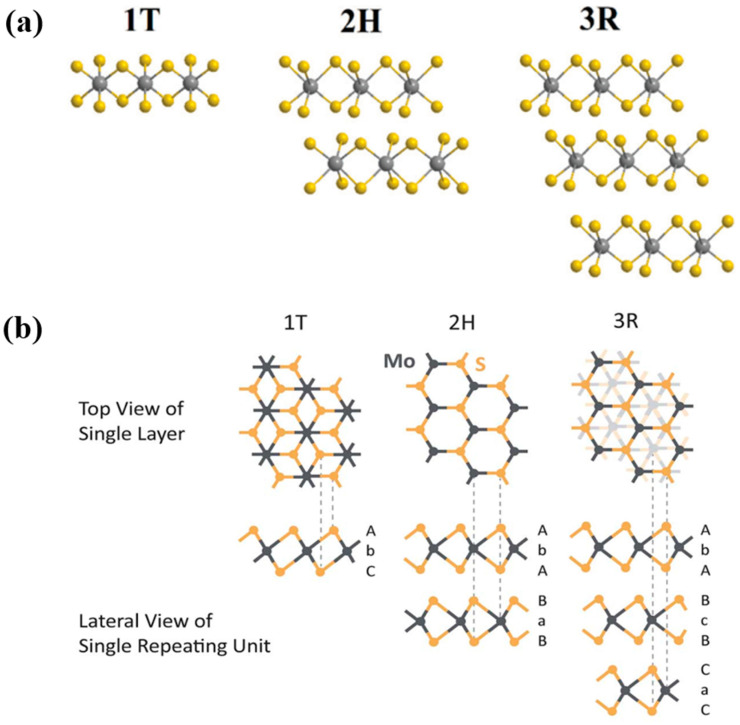
(**a**) Polytype structure of TMDs (1T, 2H, and 3R). Reprinted with permission from Coogan et al. [61] Copyright 2021, Royal Society of Chemistry. (**b**) Polytype structure of MoS_2_. Reprinted with permission from Song et al. [62] Copyright 2015, Royal Society of Chemistry.

The chemical formula of TMDs is MX_2_, where M is the transition metal from groups 4–10 in the periodic table and X is the chalcogen, as illustrated in Figure 2a. In general, TMD materials with transition metals from groups 4–7 have a layered structure, whereas some transition metals from groups 8–10, such as pyrite, have a non-layered structure [39]. The atoms in layered MX_2_ are arranged as polytypes with a transition metal atom M surrounded by six chalcogen X atoms (Figure 2b). In a TMD monolayer (basal plane), strong covalent bonds between the transition metal and chalcogen result in the formation of stacking polytypes (stacking order) and polymorphs (metal coordination geometry). As shown in Figure 3a, the TMD structures have typical configurations such as 1T, 2H, and 3R, which indicate one (1), two (2), and three (3) layers per stacked cell unit in tetragonal (T), hexagonal (H), and rhombohedral (R) phases, respectively [53,57,60,61]. For example, MoS_2_ has all the three polytypes with a regular layered structure consisting of chalcogen atoms surrounding Mo transition metal atoms (as shown in Figure 3b) [62]. It is well-known that 1T-MoS_2_ is a metastable metal phase, whereas 2H-MoS_2_ and 3R-MoS_2_ are semiconductor phases with thermodynamic stability. In particular, 1T- and 2H-MoS_2_ are different in terms of the horizontal movement of one of the two sulfur planes. The intercalation of lithium ions (Li^+^ ions) can induce the 2H-to-1T phase transition [63].

While the electronic structure of graphene is based on the hybridization of s and p orbitals, the electronic properties of TMDs are determined by the electrons filled in the d orbitals of the transition metal. Although graphene and TMDs exhibit structural similarities, the electrical properties of TMDs are determined by the number of electrons in their non-bonding d orbitals, as well as the geometrical coordination of the transition metal atoms [64]. The degree of electron filling in the d orbital significantly affects the electrical properties of TMDs, where a partially filled d orbital imparts metallic properties, whereas a completely filled d orbital leads to a semiconducting behavior [39]. As a result, transition metal atoms have a greater influence on the electronic structure of TMDs than chalcogen atoms. 

### 2.2. Advantages and Challenges of TMDs

**Figure 4:**
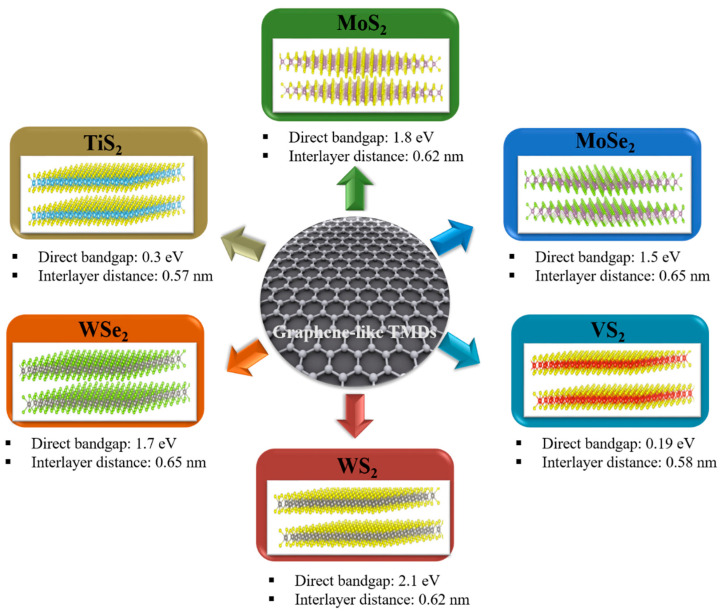
Direct bandgap and interlayer distance of various types of TMD.

Inspired by the great success of graphene, many two-dimensional (2D) materials with unique physical and chemical properties have recently gained significant attention [65,66,67,68]. In particular, layered TMDs have shown immense prospects for implementation in energy storage, catalysis, photonics, etc. [42,69,70]. Because of their large surface-to-volume ratio, which allows significantly increased interaction between the active material and electrolyte, graphene-like 2D TMDs are highly advantageous for battery applications [71,72]. Owing to the weak van der Waals (vdW) force, the ions can diffuse rapidly through the interlayer gap of MX_2_ layer. The large interlayer distance between the MX_2_ layer, in particular, makes it possible to accommodate multivalent ions such as Zn^2+^, Mg^2+^, Al^3+^, and Ca^2+^ [51,72,73].The interlayer distance and bandgaps of various TMDs commonly used in AMMIBs are shown in Figure 4.

Despite these benefits, the poor rate performance and cyclic stability of TMDs due to their low ionic conductance and large volume expansion limit their application as cathode materials in AMMIBs. Furthermore, the irreversible side effects during the charging and discharging processes decrease the coulombic efficiency of TMDs [59]. These disadvantages severely limit the application of TMDs as cathode materials for AMMIBs. For example, MoS_2_ is considered as a suitable cathode for Zn^2+^ storage owing to its large interlayer distance (0.62 nm) compared to its much smaller size (0.15 nm). Nevertheless, as reported by Liu et al., bulk MoS_2_ delivers a specific capacity of only 40 mAh g^−1^ because of the lack of clear redox peaks during electrochemical reactions [24]. This clearly shows that the interlayer distance of MoS_2_ is not the only factor that guarantees the high performance of ZIBs. The more important issue is the efficient ion adsorption on the electrode, which influences the electromigration characteristics of multivalent ions (M^z+^) in the aqueous state. Hence, the ionic radius in the hydrated state (i.e., M^z+^(H_2_O)_n_) is more important than the general ionic radius (i.e., M^z+^). In practice, the radius of hydrated Zn^2+^ ions (0.404–0.430 nm) can obstruct their absorption into the minimum interlayer distances that the host TMD material can accommodate [74]. Another important issue affecting the performance of TMD cathode materials is the variation in their structural features during the electrochemical reaction. Typically, the conductivity retained by the 1T MoS_2_ phase is 10^7^ times higher than that retained by the 2H MoS_2_ phase. In addition, while the 1T MoS_2_ phase is hydrophilic, the 2H MoS_2_ phase is hydrophobic. These different features of 1T and 2H MoS_2_ affect the adsorption and diffusion of multivalent ions in water, which eventually results in their distinct electrochemical performance in rechargeable aqueous ZIBs [75]. In addition to these fundamental aspects, the following section discusses the recent advancements of TMD cathode materials for AMMIBs.

## 3. TMD Cathode Materials for Multivalent Metal-Ion Batteries (MMIBs)

### 3.1. Zinc-Ion Batteries (ZIBs)

**Figure 5:**
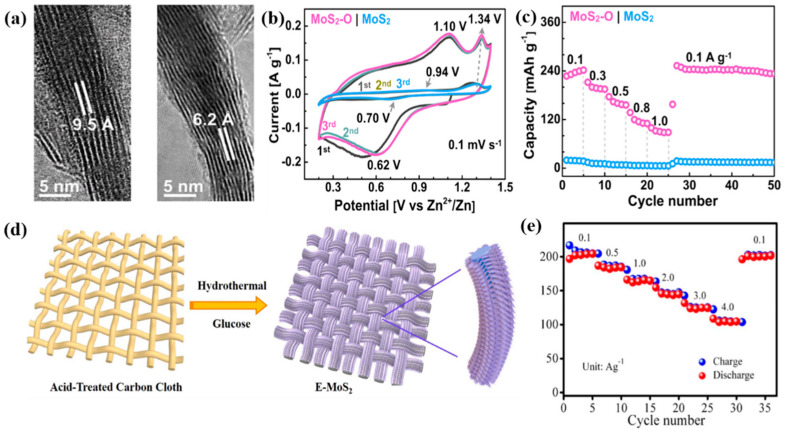
(**a**) Transmission electron microscopy (TEM) image of MoS_2_ (left:MoS_2_ with oxygen incorporation, right: bulk MoS_2_). (**b**) cyclic voltammetry curves of MoS_2_-O (pink) and bulk MoS_2_ (light blue) at a scan rate of 0.1 mV s^−1^. (**c**) Rate capability of MoS_2_-O and bulk MoS_2_ at various current densities. Reprinted with permission from Liang et al. [77] Copyright 2019, American Chemical Society. (**d**) Illustration for the preparation of E-MoS_2_. (**e**) Rate capability of E-MoS_2_ at various current densities. Reprinted with permission from Li et al. [78] Copyright 2018, Elsevier B.V.

As can be observed from Table 1, among the different types of rechargeable metal-ion batteries, ZIBs possess the following appealing characteristics: (i) high abundance and low cost of zinc, facilitating their mass production, (ii) high redox potential (−0.763V vs. standard hydrogen electrode (SHE)), (iii) high theoretical volumetric energy density of zinc anodes (5855 mAh cm^−1^), and (iv) high safety. These features make zinc ions suitable for use in both aqueous and non-aqueous batteries [23,24,76]. Recently, aqueous ZIBs (AZIBs) have gained considerable attention because of their safety, low cost, and environmental friendliness. In particular, the ionic conductance of Zn^2+^ in water (1000 mS cm^−1^) is much higher than that in organic solvents (1–10 mS cm^−1^). Nevertheless, Zn^2+^ ions easily coordinate with six water molecules, making the diffusion of Zn^2+^ into the electrode more difficult. As a result, since Liu et al. [24] first reported the poor diffusion of Zn^2+^ ions into bulk MoS_2_, there have been a limited number of studies on this material with satisfactory results. For instance, Liang et al. proposed an increase in the interlayer distance and hydrophilicity of MoS_2_ via oxygen incorporation using a hydrothermal method to enhance the inherent diffusion of Zn^2+^ into the MoS_2_ layers [77]. The pristine MoS_2_ layers were activated by adding oxygen (replacing sulfur with oxygen) because of its smaller atomic radius (48 pm) than that of S (88 pm). The vdW interaction between the interlayers was reduced by the formation of the Mo–O bond; thus, the interlayer distance widened from 6.2 to 9.5 Å (Figure 5a). As a result, the presence of a small amount of oxygen (5%) in the layered MoS_2_ increased the Zn^2+^ diffusion coefficient by three orders of magnitude. Consequently, the activated MoS_2_ had a high Zn^2+^ storage capacity of 232 mAh g^−1^ (increased by approximately 10 times after the oxygen activation), and showed pronounced redox peaks as compared to the unmodified counterpart (Figure 5b,c). Li et al. also reported the expansion of the interlayer distance through its vertical alignment on a carbon fiber fabric by hydrothermal reaction (Figure 5d) [78]. This novel structure made the assembly suitable for Zn^2+^ diffusion because of the following reasons: (i) The interfacial contact between MoS_2_ and the electrolyte was improved through the 3D network of the carbon fibers, which facilitated the formation of Zn^2+^ diffusion pathways. (ii) The extended distance between the layers reduced the ionic diffusion resistance, thereby enhancing the reaction kinetics and reducing the energy barrier for Zn^2+^ diffusion. (iii) The carbon fibers derived from glucose facilitated the interaction between MoS_2_ and the carbon-based substrate through a good conducting network. As such, the expanded MoS_2_ delivered a discharge capacity of 202.6 mAh g^−1^ and excellent cycling performance (capacity retention of 98.6% after 600 cycles) along with a high rate capability (Figure 5e). They proposed the following Zn^2+^ storage mechanism: (1) at the cathode: xZn^2+^ + x2e^−^ +MoS_2_ ↔ Zn_x_MoS_2_; (2) at the anode: xZn + x2e^−^ ↔ xZn.

On a different note, Liu et al. reported that the proportion of the 1T MoS_2_ phase affects the performance of ZIBs [75]. The 1T MoS_2_ phase content was controlled via a hydrothermal reaction over the temperature range of 140−220 °C. At 160 °C, the percentage of 1T MoS_2_ was as high as 70%, which reduced significantly to 0.4% at 220 °C. The 1T MoS_2_ phase showed a significantly lower energy barrier for Zn^2+^ than the 2H MoS_2_ phase, further facilitating the diffusion of Zn^2+^. Consequently, the MoS_2_ with a high 1T phase content exhibited excellent electrochemical performance with a high capacity retention of 98.1% and a coulombic efficiency of ~100% after 400 cycles. These results suggest that the 1T MoS_2_ phase can effectively accelerate charge transfer because of its lower diffusion energy barrier for Zn^2+^ than that of the 2H MoS_2_ phase for the same interlayer distance. This study demonstrated the importance of phase control for MoS_2_ which can influence the electron density in TMDs, eventually affecting the battery performance. Understanding the exact mechanism and the conditions regarding how different phases can trigger the phase transition can provide new opportunities to realize high-performance TMD electrodes in the future.

**Figure 6:**
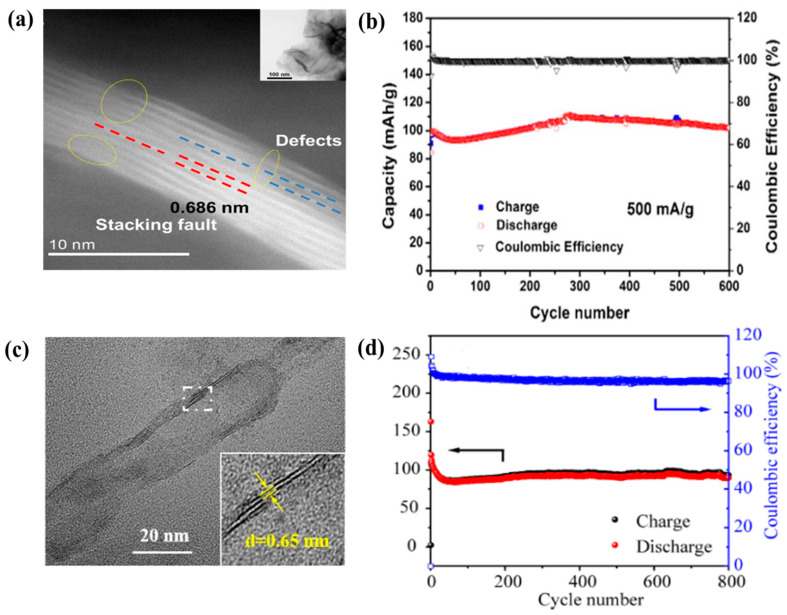
(**a**) TEM image of defect-rich MoS_2_ nanosheets, (**b**) cyclic performance of defect-rich MoS_2_ nanosheets at 200 mA g^−1^. Reprinted with permission from Xu et al. [79] Copyright 2018, Elsevier B.V. (**c**) TEM image of tubular MoS_2_, (**d**) cyclic performance of tubular MoS_2_ at 500 mA g^−1^. Reprinted with permission from Yang et al. [80] Copyright 2020, ESG Publication.

Xu et al. applied defect engineering to prepare defect-rich MoS_2_ nanosheets via a facile hydrothermal process accompanied by heat treatment [79]. The introduction of defects into nanomaterials can be promising strategies because they can store more foreign ions and enhance the electrochemical performance of these batteries. From many theoretical predictions, researchers have found that defects can increase the active sites in the electrode. The presence of defects increases the surface energy of the electrode and provides new active sites, which promotes the ion adsorption and increase the capacity. Numerous edge sites and vacancies were created in these nanosheets in a controlled manner. Indeed, these defects facilitated the diffusion of Zn^2+^ and significantly improved the reversibility of the activated MoS_2_ as compared to its pristine counterpart. Additionally, the defects in MoS_2_ could increase its interaction with Zn^2+^, and at the same time, could extend its interlayer distance. Figure 6a shows the (100) plane stacking fault and disordered atomic disposition on the surface of the material. The inefficient plane stacking resulted in a significant increase in the edge spacing and a slightly increased interlayer distance (0.686 nm). In addition, a disturbance of the atoms caused cracking of the planes, leading to the formation of additional edges. The defect-rich MoS_2_ accelerated the diffusion of Zn^2+^ owing to the formation of new transport pathways at the numerous edges and defects. As a result, a satisfactory discharge capacity of 102.4 mAh g^−1^ was obtained after 600 cycles at 500 mA g^−1^, demonstrating the promising energy storage ability of MoS_2_ (Figure 6b). Yang et al. reported novel MoS_2_ nanosheets with a porous tubular structure prepared via template-assisted thermal decomposition [80]. As can be observed from the transmission electron microscopy (TEM) image (Figure 6c), the MoS_2_ nanotube exhibited a layered structure with a interlayer distance of 0.65 nm. The tubular MoS_2_ showed the following merits for Zn^2+^ diffusion: (i) the formation of voids in the structure of the MoS_2_ nanosheets (the ratio of Mo and S atoms of 1:1.4) improved its ability to accommodate Zn^2+^; (ii) the tubular structure of the MoS_2_ nanosheets enhanced their electrolyte uptake and ion diffusion, which facilitated electron transport during the charge/discharge process. Thus, the novel MoS_2_ nanosheets exhibited a good discharge capacity of 146.2 mAh g^−1^ at 200 mA g^−1^ and excellent cycling performance with 74.0% capacity retention after 800 cycles.

**Figure 7:**
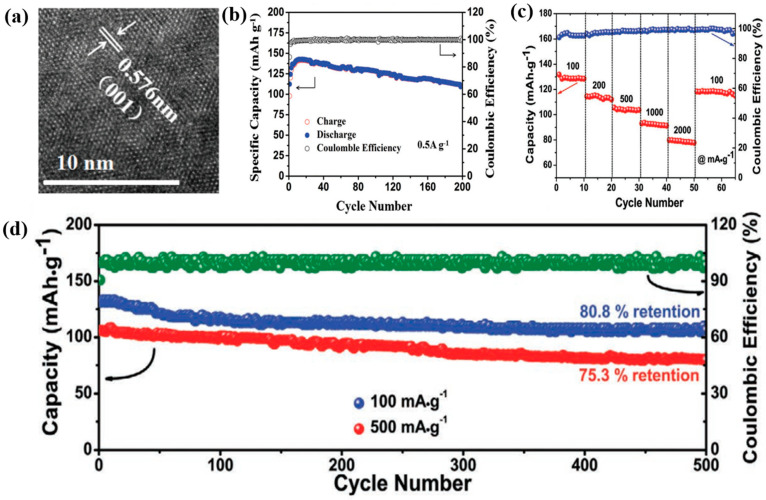
(**a**) TEM image of VS_2_ nanosheets, (**b**) cyclic performance of VS_2_ nanosheets at 500 mA g^−1^. Reprinted with permission from He et al. [81] Copyright 2017,WILEY-VCH Verlag GmbH and Co. KGaA, Weinheim. (**c**) Rate capability of VS_4_, (**d**) cyclic performance of VSe_2_ nanosheets at 100 and 500 mA g^−1^. Reprinted with permission from Wu et al. [83] Copyright 2020, Wiley-VCH GmbH.

In addition to MoS_2_, vanadium-based TMDs have also received considerable attention as promising materials for ZIB cathodes. For instance, He et al. prepared vanadium disulfide (VS_2_) nanosheets via a facile hydrothermal reaction for the first time [81]. The layered VS_2_ with high specific capacity and cyclic stability showed a huge potential for application in ZIBs. As shown in Figure 7a, the interlayer distance of the prepared VS_2_ was as high as 0.576 nm, which facilitated the diffusion of Zn^2+^. The as-prepared VS_2_ exhibited a high discharge capacity of 190.3 mAh g^−1^ at 50 mA g^−1^ and showed good cyclic stability (retained 98.0% of the initial capacity after 200 cycles) (Figure 7b). They proposed the occurrence of the following electrochemical reactions: (1) at the cathode, VS_2_+ 0.09Zn^2+^+ 0.18e^−^ ↔ Zn_0.09_VS_2_, and Zn_0.09_VS_2_+ 0.14Zn^2+^ + 0.28e^−^ ↔ Zn_0.23_VS_2_; (2) at the anode: Zn^2+^ + 2e^−^ ↔ Zn.

Jiao et al. prepared a hierarchical 1T VS_2_ directly on a stainless steel mesh substrate (VS_2_@SS) without inactive materials such as binder and conductive additives to increase the active material content [82]. The open-flower structure of VS_2_ improved the interaction between the active material and electrolyte, which contributed to the favorable ion and electron transport. The free-standing VS_2_@SS electrodes showed several outstanding advantages: the VS_2_ flower structure was suitable for accommodating volume expansion, thereby reducing the chances of electrode degradation. In addition, the layered structure of the VS_2_ flower (interlayer spacing of 0.58 nm) was favorable for ion transport because it interacted well with the electrolyte and shortened the Zn^2+^ diffusion length. In general, the maximum electrochemical performance is achieved when the loading amount of the active material is small (below 3 mg cm^−2^). However, in this work, even when the active material loading was 4–5 mg cm^−2^, the VS_2_@SS electrode showed high discharge capacity (198 mAh g^−1^ at 50 mA g^−1^) and excellent cycling performance over 2000 cycles at 2000 mA g^−1^. With the increase in the mass loading, the thickness of electrode increases. An excessively high electrode thickness is likely to be fractured or even delaminated from the current collector during electrochemical reactions, resulting in the cycling instability. Moreover, the presence of binder and conductive additives in the conventional slurry-coated method reduces the gravimetric/volumetric energy density of the electrode. Therefore, the novel structure of VS_2_@SS without binder and conductive additives could resolve this issue. The good cyclic stability of VS_2_@SS was still achieved (90% retention for 600 cycles) even when the active material loading was increased to be 11 mg cm^−2^ in this study.

Wu et al. synthesized ultrathin vanadium diselenide (VSe_2_) nanosheets via a wet-chemical route [83]. The aqueous ZIB exhibited excellent rate capability with the discharge capacities of 131.8, 114.6, 105.2, 93.9, and 79.5 mAh g^–1^ at the current densities of 100, 200, 500, 1000, and 2000 mA g^−1^, respectively. When the current density returned to 100 mA g^−1^, the recovered capacity was 118.4 mAh g^−1^ (Figure 7c). In addition, the VSe_2_ nanosheets showed good specific capacity (131.8 mAh g^−1^ at 100 mA g^−1^) and cycling performance (retained capacity of 80.8% after 500 cycles) (Figure 7d). The excellent performance of the VSe_2_ nanosheet cathode could be attributed to the following reasons: (i) reversible intercalation/de-intercalation of Zn^2+^; (ii) rapid Zn^2+^ diffusion dynamics in the ultrathin 2D structures; (iii) metallic features of VSe_2_, which promoted its Zn^2+^ storage kinetics; and (iv) structural robustness during long-term cycling. The possible Zn^2+^ storage mechanism is as follows: (1) at the cathode: VSe_2_+ 0.23Zn^2+^+ 0.46e^−^ ↔ Zn_0.23_VSe_2_, and Zn_0.23_VSe_2_+ 0.17Zn^2+^+ 0.34e^−^ ↔ Zn_0.4_VSe_2_; (2) at the anode: 0.4 Zn ↔ 0.4Zn^2+^ + 0.8e^−^.

Zhu et al. prepared a chain crystal framework of vanadium tetrasulfide (VS_4_) via a hydrothermal method as a cathode material for ZIBs [84]. With a loosely stacked structure formed by the atomic chains of VS_4_ bonded by a weak vdW force, VS_4_ was considered as a potential cathode for ZIBs. The Zn^2+^ reaction mechanism was explained as follows: (1) at the cathode, VS_4_+ 0.49Zn^2+^+ 0.98e^−^ ↔ Zn_0.49_VS_4_, and Zn_0.49_VS_4_+ 0.54Zn^2+^+ 1.08e^−^ ↔ Zn_1.03_VS_4_; (2) at the anode: Zn ↔ Zn^2+^ + 2e^−^. As a result, the maximum capacity reached 110 mAh g^−1^, and high capacity was obtained even after 500 cycles, which is favorable for practical applications. The electrochemical performances of the recently reported TMD cathodes for ZIBs are summarized in Table 2.

### 3.2. Magnesium-Ion Batteries (MIBs)

Over the past few years, MIBs have been extensively studied owing to their environmentally friendliness, high abundance of Mg, high energy density, low reduction potential, and virtually non-dendrite formation [85]. The energy storage mechanism of MIBs is similar to that of ZIBs: intercalation and conversion processes. Although the ionic radius (0.72Å) and hydrated ionic radius of Mg^2+^(4.28 Å) are similar to those of Li^+^ (ionic radius of 0.76 Å and hydrated ionic radius of 4.3 Å of Li^+^), most cathode materials for LIBs are not favorable for MIBs owing to the high charge density of Mg^2^^+^ [86]. Cathode materials for MIBs should exhibit high reversible capacities under adequate operating voltages. The kinetics of Mg^2+^ diffusion through the cathode material is usually slow because of the high valence of Mg^2+^ ions, redistribution of divalent cations in the host material, and strong ionic interactions. Various strategies have been reported to overcome these limitations. 

**Figure 8:**
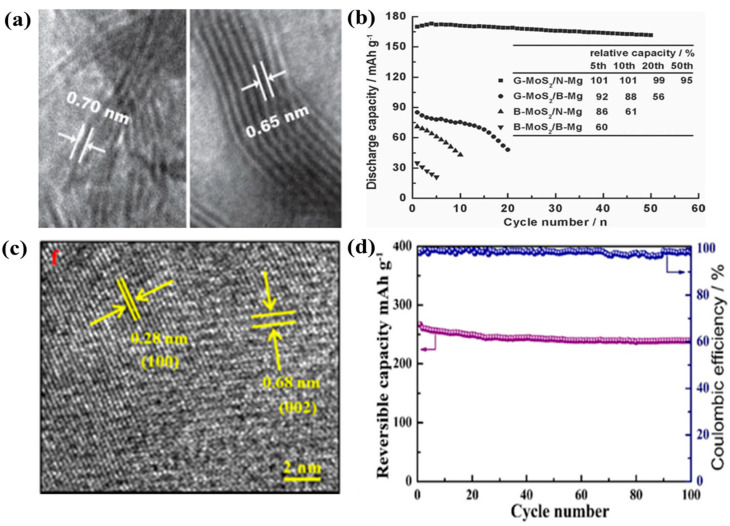
(**a**) TEM image of G-MoS_2_ nanosheet, (**b**) cyclic performance of G-MoS_2_ and B-MoS_2_ with Mg nanoparticle (N-Mg) and bulk Mg (B-Mg) anodes at 20 mA g^−1^. Reprinted with permission from Liang et al. [88] Copyright 2011, WILEY-VCH Verlag GmbH and Co. KGaA, Weinheim.(**c**) TEM image of WSe_2_ nanosheets, (**d**) cyclic performance of WSe_2_ nanosheets at 50 mA g^−1^. Reprinted with permission from Xu et al. [93] Copyright 2020, Elsevier Inc.

Yang et al. carried out first-principle studies and proposed MoS_2_ with a zigzag structure as a favorable cathode for rechargeable MIBs [87]. This study showed the detailed diffusion pathways for Mg^2+^ in the MoS_2_ nanoribbons. The specific diffusion path of Mg^2+^ was shown to be through a nearest-neighbor H site in a zigzag manner between two adjacent T sites in MoS_2_. This pathway in the MoS_2_ nanoribbons was different from the conventional pathways of graphene nanoribbons, where Mg^2+^ ions undergo hopping between the intralayer hollow sites across the C–C bridge. The maximum theoretical capacity of a MoS_2_ nanoribbon (width of 5 nm) that can accommodate six Mg atoms was predicted to be 232.2 mAh g^−1^. Liang et al. synthesized highly exfoliated, graphene-like MoS_2_ (G-MoS_2_) as a cathode material for MIBs through a solvothermal process [88]. The synthesis of G-MoS_2_ was from solvothermal reaction between MoO_3_ and thioacetamide with pyridine as a solvent. The nature of few layer MoS_2_ was confirmed by XRD which showed significantly reduced intensity of (002) peak for G-MoS_2_ compared with bulk-MoS_2_ (B-MoS_2_). In addition, the interlayer distance of the prepared G-MoS_2_ was 0.65–0.70 nm, which is larger than that of B-MoS_2_ (0.63 nm) (Figure 8a). Apart from cathode side, the anode part was also studied; the ultra-small Mg nanoparticles (N-Mg, diameter of ~2.5 nm) was prepared by ionic liquid-assisted chemical reduction and compared with bulk Mg (B-Mg). The full cell performance of four different cells (G-MoS_2_/N-Mg, G-MoS_2_/B-Mg, B-MoS_2_/N-Mg, and B-MoS_2_/B-Mg) was systematically compared (Figure 8b). The results showed significantly improved cyclic performance for G-MoS_2_/N-Mg cell in terms of specific capacity and cyclic stability. The measured Mg storage capacity of MoS_2_ was estimated to be 170 mAh g^−1^ after 50 cycles. It was noted that this capacity was 24% lower than its theoretical capacity (232.2 mAh g^−1^), which probably originated in the partial restacking of the MoS_2_ layers and the intercalation of Mg only on one side of the MoS_2_ layer. The high performance of G-MoS_2_/N-Mg cell was attributed to the reduction in the passive film on the surface of N-Mg that increases the diffusion coefficient and increased available intercalation sites in g-MoS_2_. Liu et al. fabricated MoS_2_/C microspheres with a sandwich structure via a hydrothermal process and heat treatment [89]. The carbon formed by the hydrothermal glucose carbonization improved the ionic conductance and extended the interlayer spacing, facilitating the diffusion of Mg^2+^ and making the process reversible. In addition, the graphene-like MoS_2_/C structure promoted the access of Mg^2+^ ions to the electrolyte, facilitating Mg^2+^ transport. As a result, the sandwiched MoS_2_/C delivered a discharge capacity of 118.8 mAh g^−1^ after 20 cycles at 50 mA g^−1^ and exhibited excellent cycling stability as compared to bulk MoS_2_. Truong et al. reported the rapid exfoliation of MoS_2_ and molybdenum diselenide (MoSe_2_) nanosheets using the supercritical fluid (SCF) method [90]. SCF exfoliation is a facile and fast process used to produce high-quality TMD nanosheets (few-layer). They applied this process to MoS_2_ and MoSe_2_ and obtained few-layer (1–10 layers) nanosheets with hexagonal structures (2H stacking sequence). The structure was precisely observed using atomic-resolution high-angle annular dark-field imaging. When used as MIB electrodes, the MoS_2_ and MoSe_2_ nanosheets delivered the specific capacities of 81 mAh g^−1^ after 10 cycles and 55 mAh g^−1^ after five cycles at 0.02 A g^−1^, respectively, which are superior to those of pristine MoS_2_ and MoSe_2_. 

Mao et al. studied layered MX_2_ (M = Ti, V; X = O, S, Se) as a model to examine how different chalcogen species affect the Mg intercalation dynamics of MIBs [91]. TiSe_2_ showed the best electrochemical performance among all the samples investigated owing to the following factors: (i) the interlayer spacing of TiSe_2_ was greater than the size of Mg^2+^; (ii) the vdW interaction between the basal planes of TiSe_2_ was very weak, which facilitated the diffusion of Mg^2+^ into the layers; (iii) the electronic conductivity of TiSe_2_ was higher than those of its counterparts. In addition, the authors proposed further modification methods to improve the intercalation kinetics of the compound, including: (i) using anions with less electronegativity, (ii) diminishing the electrostatic interaction between Mg^2+^ and the host (e.g., incorporation of monovalent anions on the host materials); (iii) developing an open-tunnel structure for the MX_2_ to redistribute the charge and electric conductivity. This report provides guidelines for choosing and designing high-performance cathodes with rapid dynamics for MIBs. Gu et al. developed a MIB with a micrometer-sized TiSe_2_ cathode operated at room temperature [92]. They found that the crucial factor that improves the reversible Mg^2+^-intercalation/deintercalation is charge displacement in the metal-binding units, which is induced by the strong d-p orbital hybridization in TiSe_2_. In the case of selenides, the highly overlapped d and p orbitals promote d-p orbital hybridization because of the larger 4p-orbital dimensions of selenides than those of oxides or sulfides. Furthermore, 2D ion-conducting channels in the gap between the basal planes of TiSe_2_ reduce the coulombic repulsion among the Mg ions. This study has demonstrated the importance of d-p orbital hybridization between transition metal atom and the chalcogen atom in TMDs. The strong d-p orbital hybridization induced by their close energy levels might be one of the key factors, which improves the reversible intercalation/deintercalation of Mg^2+^. It will be interesting whether such concept can be applicable to other types of multivalent ions (Zn^2+^ and Al^3+^) for the TMD electrodes. Xu et al. employed a simple one-step hydrothermal method to synthesize a flower-like tungsten diselenide (WSe_2_) nanosheet [93]. Figure 8c shows the lattice spacings of 0.68 and 0.28 nm, which correspond to the d-spacing of the (002) and (100) crystal plane of WSe_2_, respectively. There have been only a limited number of studies utilizing WSe_2_ for MIBs due to its low conductivity and structural instability that leads to the rapid degradation during electrochemical reactions. However, in this work, they overcame these by developing a novel structure (i.e., orderly flower structure) that effectively increased the contact area between electrode and electrolyte and allowed abundant ion channels through highly connected three-dimensional nanostructure. The WSe_2_ cathode showed a high reversible capacity of 265 mAh g^−1^, excellent cycling performance with a capacity retention of 90% after 100 cycles at 50 mA g^−1^, and excellent rate capability with 70% capacity retention even at 500 mA g^−1^ (Figure 8d). The following Mg^2+^ storage mechanism was proposed: (1) at the cathode: 6WSe_2_+ 4Mg^2+^+ 8e^−^ ↔ Mg_4_W_6_Se_12_; (2) at the anode: 4Mg ↔ 4Mg^2+^+ 8e^−^. The hydrothermal method is one of the common processes for the synthesis of novel TMD structures. The property of TMD is strongly related to its phase, size, morphology, and crystallinity. These features can be controlled by rational design and careful tuning of the hydrothermal process conditions. Therefore, it is important to understand the general mechanism of the hydrothermal growth of TMD (e.g., how parameters such as the precursor, substrate, additive, temperature, reaction time, and solvent affect the growth of TMD materials). The electrochemical performances of the recently reported MD-based MIB cathodes are summarized in Table 3. Based on Table 3, it can be clearly seen that the new structure design of TMD is one of the important strategies to create high-performance cathodes in MIBs. To prevent the restacking of TMD nanosheets, one of the most effective approaches is to prepare layered TMD nanomaterials with a hierarchical structure. In this hierarchical structure, the high surface-to-volume ratio offers abundant electrochemical active sites for ion storage, shortens the diffusion distance of Mg^2+^ ions, and alleviates the volume change of layered TMD nanomaterials by the presence of large voids during the repetitive charge/discharge processes. In this context, the hierarchical WSe_2_ nanoflower has been demonstrated as a promising material in terms of the specific capacity and capacity retention (Table 3) [93]. Moreover, the carbon coating of layered TMD nanomaterials is another effective strategy because it can give high electrical conductivity and good elasticity for TMD materials.

### 3.3. Aluminum-Ion Batteries (AIBs)

The concept of rechargeable AIBs was first discovered in 1970 [94]. AIBs offer many advantages such as low cost, high safety, and high electrochemical energy storage. Nevertheless, AIBs suffer from many challenges, including low ionic diffusion [95], material disintegration [96], poor durability [97,98,99,100,101,102,103], and the formation of passive oxide layers [46]. The energy storage mechanism of AIBs is similar to that of the other AMMIBs (the conversion and intercalation reactions); however, the hydrated ionic radius of Al^3+^ (4.75 Å) is larger than those of other multivalent charge carriers (Zn^2+^, Mg^2+^, and Ca^2+^). Thus, it is difficult to select a suitable cathode material for AIBs.

**Figure 9:**
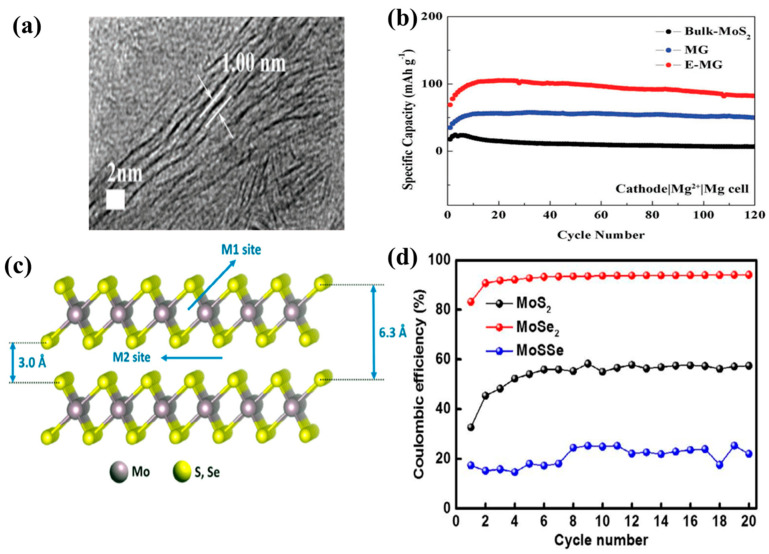
(**a**) TEM image of freestanding MoS_2_-graphene foam composite with glucose (E-MG), (**b**) cyclic performance of bulk-MoS_2_, MoS_2_-graphene foam without glucose (MG), and E-MG at 20 mA g^−1^. Reprinted with permission from Fan et al. [104] Copyright 2017, WILEY-VCH Verlag GmbH and Co. KGaA, Weinheim. (**c**) Schematic of the MoX_2_ structure (X: S, Se) with M1 and M2 site, (**d**) coulombic efficiency of MoX_2_ at 100 mA g^−1^. Reprinted with permission from Divya et al. [106] Copyright 2020, WILEY-VCH Verlag GmbH and Co. KGaA, Weinheim.

Fan et al. prepared interlayer-expanded MoS_2_ nanosheets on graphene foam via a hydrothermal process as a cathode material for AIBs [104]. They expanded the interlayer distance of the MoS_2_ nanosheets to 1.0 nm (Figure 9a), which improved the structural stability of the nanosheets and prevented large volume changes due to the facile intercalation of Al^3+^. Therefore, the diffusion barrier of Al^3+^ and ion trapping were greatly reduced, thus increasing the number of ion storage locations. The amorphous carbon used in this study was formed by heat treatment at 450 °C, which enhanced the interaction between the MoS_2_ sheets and graphene foam, resulting in the formation of a highly conductive three-dimensional (3D) structure. As a result, the MoS_2_ nanosheets exhibited a specific capacity of 105 mAh g^−1^ after 20 cycles, and the capacity decreased gradually to 87.6 mAh g^−1^ after 120 cycles at 200 mA g^−1^ (Figure 9b). Li et al. prepared MoS_2_ microspheres as a cathode material for AIBs via a simple hydrothermal process [105]. They analyzed the Al^3+^ intercalation and deintercalation sites in the MoS_2_ microspheres as the S–Mo–S bonding (A1) and vdW gap (A2) sites. During the charge insertion/deinsertion process, the A1 sites tended to lose their capacity and underwent a phase transition, whereas the A2 sites were more stable for the (de)intercalation of the Al^3+^ ions. They described the process of (de) intercalation of Al^3+^ as follows: (i) the discharge process: Al^3+^ initially intercalated at the A1 sites, then continued to intercalate at the A2 sites; (ii) charge process: Al^3+^ first deintercalated from the A2 sites and finally from the A1 sites of the cathode. The high electrostatic interaction between Al^3+^ and S^2-^ anionic network in S-Mo-S (A1 site) led to the electrochemical polarization of MoS_2_, which was not favorable for the reversible intercalation/deintercalation of Al^3+^. This electrostatic interaction causes loss in capacity and phase transition due to high electrochemical polarization of MoS_2_. On the other hand, Al^3+^ ions adsorbed at A2 site (constructed by the van der Walls force with a small electrostatic effect) tend to undergo more reversible intercalation/deintercalation. As a result, the AIB exhibited the excellent discharge capacities of 253.6 and 66.7 mA h g^−1^ at 20 and 40 mA g^−1^, respectively, after 100 cycles. Similar results were reported by Divya et al. [106] Al^3+^ intercalated into two vacant sites, M1 and M2, where M1 is the X site in X-Mo-X atoms (X: S, Se, and SSe) and M2 represents the interlayer distance of MoX_2_ (Figure 9c). The phase transformation after the first few intercalations/deintercalations led to the accommodation of more Al^3+^ ions, resulting in a higher capacity than that of the original MoS_2_. On the other hand, this 2H-to-1T structural change led to potential deformation and disorder, resulting in an unstable capacity depending on the X atoms in MoX_2_. As a result, while MoSe_2_ showed a high coulombic efficiency of 95% at the current density of 100 mA g^−1^, the coulombic efficiencies of MoS_2_ and MoSSe were much lower (Figure 9d). 

Wu et al. prepared VS_2_ nanosheets as a highly-efficient cathode material for AIBs by physically depositing a coating on graphene (G-VS_2_) [107]. Owing to the synergistic effect of the modified layered VS_2_ and graphene (layered spacing of 5.75 Å), the AIB with G-VS_2_ showed significantly improved electrochemical performance as compared to that with pristine VS_2_. Owing to its highly stable framework, G-VS_2_ provided a good support for ion diffusion and improved electron transport properties. As a result, G-VS_2_ delivered a discharge capacity of 50 mAh g^−1^ at 100 mA g^−1^ with a coulombic efficiency of ~100% after 50 cycles (vs. 22 mAh g^−1^ of pristine VS_2_ under the same conditions). The Al^3+^ storage mechanism of G-VS_2_ is as follows: (1) at the cathode: VS_2_ + xAl^3+^ + 3xe^−^ ↔ Al_x_VS_2_; (2) at the anode: Al + AlCl_4_^−^ ↔ Al_2_Cl_7_^−^.

**Figure 10:**
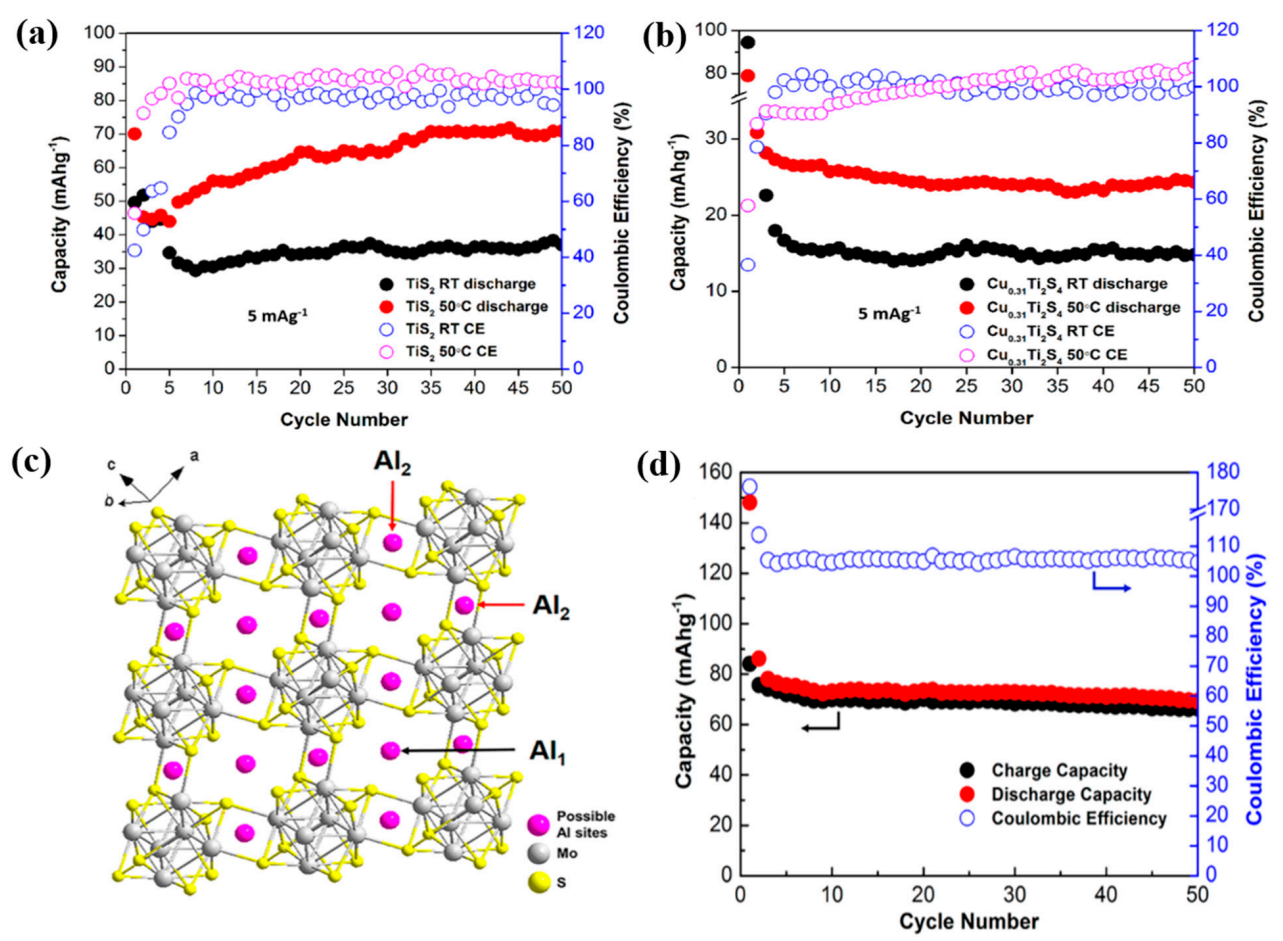
Cyclic performance of (**a**) TiS_2_ and (**b**) Cu_0.31_Ti_2_S_4_ at room temperature and 50 °C at 5 mA g^−1^. Reprinted with permission from Geng et al. [108] Copyright 2017, American Chemical Society. (**c**) Schematic structure of Al insertion sites in Mo_6_S_8_, (**d**) cyclic performance of Mo_6_S_8_. Reprinted with permission from Geng et al. [109] Copyright 2015, American Chemical Society.

Geng et al. investigated the insertion/extraction behavior of Al^3+^ in layered titanium disulfide (TiS_2_) and spinel-based cubic Cu_0.31_Ti_2_S_4_ in AIBs [108]. In their work, the Al^3+^ ions occupied mainly the octahedral sites in the layered TiS_2_ owing to the less adaptive nature of Al^3+^ ions in the layered TiS_2_. The major obstacle for the facile intercalation/deintercalation of Al^3+^ ions was associated with the Al^3+^ diffusion coefficient, as revealed by galvanostatic intermittent titration analysis results. The strong coulombic interaction between the Al^3+^ ions and anionic sulfide sites increased the energy barrier for Al^3+^ diffusion. This effect was more pronounced in the spinel-based cubic Cu_0.31_Ti_2_S_4_ than in the layered TiS_2._ As a result, the discharge capacity of the layered TiS_2_ at 50 °C was considerably higher than that at room temperature. The capacity of the layered TiS_2_ increased gradually after the first few cycles and stabilized at approximately 70 mAh g^−1^, whereas the discharge capacity of the cubic Cu_0.31_Ti_2_S_4_ at 50 °C was only approximately 25 mAh g^−1^ (Figure 10a,b). Geng et al. also prepared Mo_6_S_8_ particles using a precipitation procedure [109]. In this study, they not only demonstrated the good performance of Mo_6_S_8_, but elucidated the two distinct Al adsorption sites in chevrel phase Mo_6_S_8_. From Figure 10c, the larger site (Al_1_) can be visualized as a cubic center of a hexahedron with eight Mo_6_S_8_ units as the vertices, whereas the smaller site (Al_2_) can be visualized as a face centered hexahedron. Al^3+^ ions could occupy the Al_1_ position more easily than the Al_2_ position because of the strong electrostatic interaction between the Al^3+^ ions at the Al_1_ position. Al^3+^ ions could occupy the Al_1_ position more easily than the Al_2_ position because of the strong electrostatic interaction between the Al^3+^ ions at the Al_1_ position. Although the number of available sites for the Al intercalation was six on the hexahedron, the actual number of Al filled in the sites was only two (Al_2_Mo_6_S_8_) due to the strong electrostatic force from Al cation with three positive charges. The Al^3+^ storage mechanism of the Mo_6_S_8_ particles was described as follows: (1) at the cathode: 8[Al_2_Cl_7_]^−^ + 6e^−^ + Mo_6_S_8_ ↔ Al_2_Mo_6_S_8_ + 14[AlCl_4_]^−^; (2) at the anode: Al + 7[AlCl_4_]^−^ ↔ 4[Al_2_Cl_7_]^−^ +3e^−^. The capacity of Mo_6_S_8_ was rapidly stabilized after the first cycle and maintained at 70 mAh g^−1^ after 50 cycles (Figure 10d). The electrochemical performances of the recently reported MD-based AIBs cathodes are summarized in Table 4. At present, it is still challenging to select suitable cathode materials for AIBs because the hydrated ionic radius of Al^3+^ (4.75 Å) is larger than those of other multivalent charge carriers. Therefore, most researches regarding AIBs with TMDs are only in the immature stage of understanding the reversible intercalation and extraction of Al in various TMDs materials. Based on Table 4, MoS_2_ and G-VS_2_ have been demonstrated as impressive candidates for AIB cathodes in terms of specific capacity and capacity retention. Similar to other types of AMMIBs (ZIBs and MIBs), the control of MoS_2_ nanostructure (MoS_2_ on graphene foam [104] or MoS_2_ microsphere [105]) is efficient strategy in realizing good performance in AIBs. As for VS_2_, it possesses the potential due to its high theoretical capacity based on the multiple chemical oxidation states in vanadium and sulfide atom. Besides, the interlayer space of VS_2_ is 5.76 Å, which is large enough to enable the facile intercalation/de-intercalation of Al^3+^ ions. Furthermore, the strategy of VS_2_ hybridization with graphene (G-VS_2_) is one of the promising approaches to improve the performance because it can facilitate the Al^3+^ ion transport and reduce the restacking in VS_2_ cathode materials [107].

## 4. Modification Strategies for TMDs toward High-Performance Aqueous Multivalent Metal-Ion Batteries (AMMIBs)

The results discussed thus far emphasize the need to activate TMD cathodes to achieve good electrochemical performance for AMMIB applications. Although TMD materials have large interlayer spacing, their high energy barrier of multivalent ions limits their application as high-performance cathodes. Many modification strategies have been proposed to create structures that can realize the high electrochemical performance of TMD materials. In this section, we have summarized the modification strategies used to improve the structural stability of TMD materials in order to realize their AMMIB applications. The main modification strategies for TMDs include: (1) interlayer modification, (2) defect modification, (3) hybridization, and (4) phase modification.

### 4.1. Interlayer Modification

The metal ion (M^n+^) intercalation channel in a cathode crystal significantly affects its electrochemical behavior. Therefore, the adjustment of cation channels and coordination in the interlayer not only encourages the intercalation/deintercalation of M^n+^, but also enhances the electrochemical performance of the TMD cathode. The introduction of intercalants into 2D layered TMD materials is a viable strategy for enhancing their electrochemical performance [110].

Small guest intercalants (SGIs) such as ions, small molecules, and polymers can alter the interlayer spacing of TMDs [58,111,112,113,114]. Nevertheless, the following factors need to be considered for the incorporation of these small intercalants into the interlayer spacing of TMDs: (i) SGIs can occupy the active site and block the cationic pathway. This interaction may deteriorate the diffusion kinetics of the cations. Thus, a rigorous management of the quantity, geometry, functional structure, and interaction sites in the interlayer spacing should be taken into account to ensure effective cation diffusion; (ii) the integrity of SGIs in the TMD framework is crucial for the long lifespan of the electrode. Small molecules and low-valence ions are more susceptible to deintercalation from the lattices than high-molecular-weight polymers or high-valence ions; (iii) the introduction of SGIs with unnecessary mass and volume leads to a decrease in the theoretical power and energy densities of the electrode. In general, the interlayer modification of cathodic materials should be carried out carefully to ensure cationic intercalation and deintercalation without negatively affecting the electrochemical properties of the electrode [115].

Chemical vapor transport, electrochemical treatment, ion exchange, and oxide-reduction methods have been used to intercalate SGIs into TMDs [77,112]. The insertion of SGIs can modify the electron filling state in the orbital and Fermi stages of TMDs, often resulting in unusual properties such as superconductivity, charge density waves, and Peierls instability (characteristic phenomenon of one-dimensional electron-lattice systems). The increased interlayer distance is extremely effective in lowering the M^n+^ intercalation energy barrier. In view of these benefits, there have been several reports on using various SGIs to modify the interlayer spacing of TMDs. The benefits and drawbacks of intercalation strategies (intercalation of hydrophilic and carbon species and exfoliation technique) are discussed in the following sections.

#### 4.1.1. Intercalation of Hydrophilic Species into TMDs

**Figure 11:**
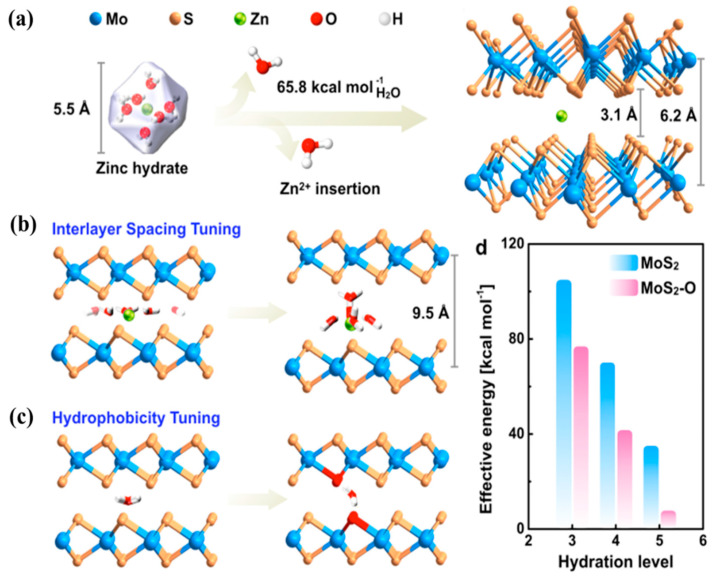
(**a**) Schematic showing the difficulty in the intercalation of Zn hydrates into bulk MoS_2_ owing to the large energy barrier between the layers, (**b**) the expanded interlayer distance that supports the diffusion of Zn^2+^, (**c**) hydrophobicity control by the Zn–H_2_O–O interaction, (**d**) theoretical energy barrier between MoS_2_ and MoS_2_–O depending on the hydration level of Zn^2+^. Reprinted with permission from Liang et al. [77] Copyright 2019, American Chemical Society.

The introduction of an oxygen atom into MoS_2_ has been suggested as an effective strategy for lowering the energy barrier of Zn^2+^, enhancing the intrinsic diffusion and facilitating structural changes in layered MoS_2_ (Figure 11). Because the interlayer distance of pristine MoS_2_ (3.1 Å) is too small, the insertion of large Zn^2+^ hydrate molecules (5.5 Å) is difficult. According to density functional theory (DFT) calculations, a 66 kcal mol^−1^ energy per coordination (Zn–O) bond is required to break a hydrated Zn^II^–OH_2_ bonds to form Zn^II^–S bonds, which is a large energetic penalty (Figure 11a). However, the energetic requirement can be significantly reduced when the interlayer distance is increased by introducing OH functional groups to form Zn–OH bonds without breaking the water molecules complexed with Zn^2+^ ion. The interlayer distance of MoS_2_-O can be increased to accommodate hydrated Zn^2+^ molecules (five water molecules complexed with Zn^2+^ to form a solvated shell, as shown in Figure 11b). In addition, MoS_2_ possesses hydrophobic properties, which make the intercalation process challenging owing to the weak interaction between Zn-H_2_O and S. To resolve this, Liang et al. used oxygen atoms to replace the sulfur atom in MoS_2_, which improved the hydrophilic properties of MoS_2_ and enhanced the interaction of Zn-H_2_O-O in it (Figure 11c). Figure 11d shows the effective energy required depending on the hydration level of Zn^2+^ cations for intercalation into MoS_2_ and MoS_2_-O. An increase in the interlayer distance of MoS_2_ favors a reduction in the number of Zn–H_2_O bonds, resulting in a reduced energy barrier. The interlayer modification method used by Liang et al. can be utilized to improve the ion storage efficiency of TMDs, creating new trends for the development of advanced materials for energy storage devices.

#### 4.1.2. Exfoliation

Liquid-phase exfoliation is a direct chemical method, which has been demonstrated to be an appropriate method for the production of industrial-scale nanosheets [116,117,118,119,120,121]. Exfoliation under slight sonification [118] or high-shear mixing [121] in stable organic solvents or water-based surfactants can be utilized to prepare TMD nanosheets. Sonification-assisted liquid-phase exfoliation in organic solvents is a facile procedure for the synthesis of TMDs with few layers. The energy required for the exfoliation process is reduced owing to the strong interaction of the solvent with the bulk materials and the solvation process. The solvents used in the exfoliation method should have high solvation power. However, the limitations of this method include sensitivity to the ambient environment, structural deformation, and changes in the electronic properties. 

**Figure 12:**
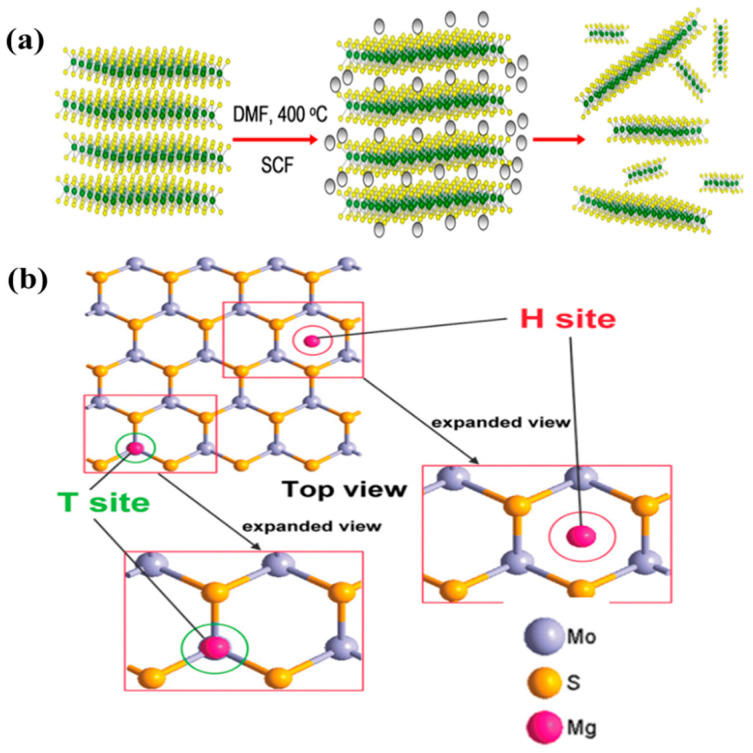
(**a**) Schematic diagram of the supercritical fluid (SCF) procedure to synthesize TMDs. Reprinted with permission from Truong et al. [90] Copyright 2017, American Chemical Society.(**b**) Illustration of two Mg adsorption positions (H and T sites) on MoS_2_ nanoribbon:. Reprinted with permission from Yang et al. [87] Copyright 2012, American Chemical Society.

Recently, the SCF method has been widely used to prepare high-quality graphene and inorganic nanosheets for industrial applications [122,123,124]. The main advantage of this method is that it can disperse the products without changing their original nature [122]. The SCF method was recently utilized to exfoliate MoS_2_ into few-layer nanosheets. Solvents used in the SCF method should have low surface tension and high diffusion coefficient, serving as a good medium to exfoliate TMDs. Truong et al. used a SCF to create few-layer (1–10) MoS_2_ and MoSe_2_ nanosheets. They demonstrated that the exfoliated nanosheets of both MoS_2_ and MoSe_2_ could retain their 2H stacking sequence during the exfoliation process (Figure 12a), [90]. Yang et al. systematically investigated the absorption of Mg on zigzag MoS_2_ nanoribbons. The results indicated that ionic bonds were formed predominantly through interactions between the guest Mg atoms and MoS_2_ nanoribbon substrate, while some covalent hybridizations still existed simultaneously to some extent. The T position at the edge of the nanoribbon was the most stable for the absorption of the guest Mg atoms. The Mg diffusion pathway was identified as two adjacent T and H positions on the zigzag MoS_2_ nanoribbons (Figure 12b). As a result, the MoS_2_ nanoribbons exhibited a maximum theoretical capacity of 223.2 mAh g^−1^ [87].

### 4.2. Defect Modification

In recent years, the defect technique has been widely used as an effective method to modify the surface properties and electronic structure of electrodes. Defect modification of the crystal structure proceeds according to the second law of thermodynamics [125,126,127]. Point defects, which play an important role in defect modification, can be divided into two categories: intrinsic and non-intrinsic defects [128,129,130]. The thermal vibration of the atomic lattice is responsible for the generation of internal defects, which do not affect the overall composition of the crystal. Schottky and Frenkel defects are examples of intrinsic defects [131,132]. Schottky defects are formed from lattice vacancies generated by the thermal vibration of atoms or ions in the crystal structure at the initial lattice position, whereas Frenkel defects are formed by the intercalation of atoms or ions into the lattice sites. Non-intrinsic defects are due to impurity atoms or ions being embedded into the lattice, and hence are also known as heteroatomic defects [133]. Point defects cause lattice distortion by disturbing the surrounding atoms, which can affect the electronic structure, chemical properties, or ionic conductance of the material, thereby regulating its electrochemical properties. The defects can be formed ranging from faults on an atomic scale that are inherent to crystallographic structures to larger defects that are introduced during fabrication process. Generally, it is difficult to control the defects caused by the fabrication process because they are non-uniform and unpredictable, leading to a serious failure during fabrication. The misapplication of the manufacturing process or lack of control at any stage may introduce defects and residual stresses that can affect the performance of the materials, making it susceptible to failure. These types of defects can include holes, cracks, segregation, inclusions, surface marks, notches and other undesirable or unintentional property changes within the material [134]. 

Defect sites can be the active, storage, and absorption sites that can immobilize the foreign ions during the reaction process. Defect modification affects the (de)intercalation of metal ions in layered materials, causing a decrease in the stress and electrostatic force between the adjacent layers, which reduces the diffusion energy barrier and promotes ion dissolution as well as charge transfer during metal ion intercalation. The main effects of defect modification in a material are as follows: (i) the ionic charge can be redistributed through defect engineering, thereby promoting the process of ion diffusion and electron transfer; (ii) the ion storage can be increased through the defect sites, leading to an increase in the capacity of the material; (iii) defect engineering also contributes to the enhanced dynamics in electrochemical reactions and phase transitions owing to the formation of multiple active sites in TMDs; (iv) defect engineering enhances the structural stability of TMD materials, making them resistant to structural damage caused by the insertion/extraction of foreign ions. As a result, the defect modification of TMDs may be a feasible approach to improve their electrochemical performance for application as ZIB cathodes. For instance, to facilitate Zn^2+^ intercalation in MoS_2_, Xu et al. used defect engineering by controlling its multiple edge and void sites [79]. The defects so formed included exposed edge sites and defects within the basal planes of MoS_2_. The defects formed were S vacancies combined with a disturbed atomic arrangement, resulting in the cracking of the basal plane and the formation of complementary edges or boundaries. These defects generated a large number of active sites in the electrode, which improved its electrochemical performance in the ZIB. Similar defect strategies have been applied in manganese oxide cathode materials (MnO_2_). Xiong et al. [135] studied the use of oxygen deficient σ-MnO_2_ as a cathode material for ZIB. Gibbs free energy of Zn^2+^ adsorption in the vicinity of oxygen deficient region can be reduced to a thermoneutral value (≈0.05 eV) by generating oxygen vacancies (OV) in the MnO_2_ lattice. This suggests that Zn^2+^ adsorption/desorption process on oxygen-deficient MnO_2_ is more reversible as compared to pristine MnO_2_. In addition, because fewer electrons are needed for Zn–O bonding in oxygen-deficient MnO_2_, more valence electrons can be contributed to the delocalized electron cloud of the material, which promotes the attainable capacity. As a result, the stable Zn/oxygen-deficient MnO_2_ battery was able to deliver one of the highest capacities of 345 mAh g^−1^ reported for a birnessite MnO_2_ system.

### 4.3. Hybridization with Carbon

Hybridization of two or more materials is a technique that combines the advantages of all the components to create novel materials with improved functionalities and electrochemical performance [136,137]. Layered TMD nanomaterials are often hybridized with other materials such as carbon, metal sulfides, metal oxides, and conductive polymers. Among these, carbon-based materials are the most commonly used materials to form hybrids with TMDs [138]. The high electrical conductivity of carbon materials accelerates the electron transport in TMDs and reduces the diffusion energy barrier, thereby improving their electrochemical performance.

**Figure 13:**
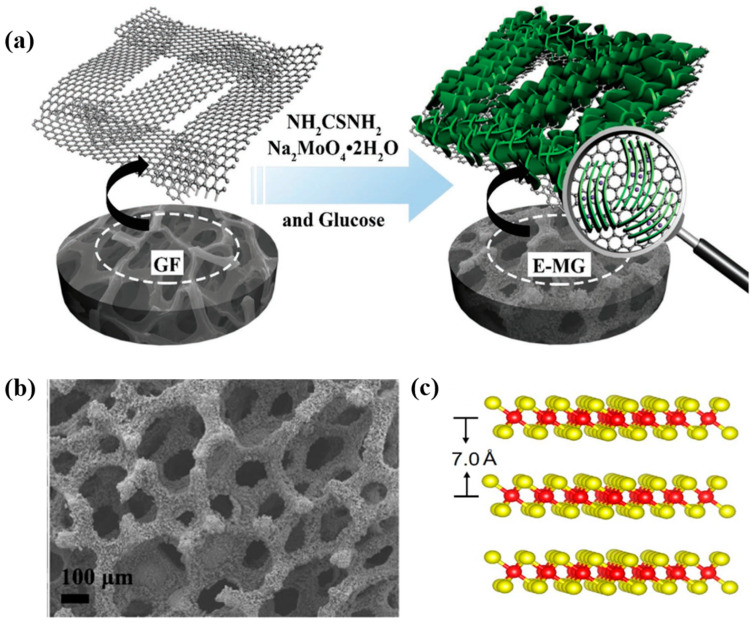
(**a**) Schematic for the synthesis of E-MG. (**b**) Scanning electron microscopy (SEM) image of free-standing MoS_2_/graphene foam. Reprinted with permission from Fan et al. [104] Copyright 2017, WILEY-VCH Verlag GmbH and Co. KGaA, Weinheim. (**c**) The atomic structure with the expanded interlayer distance of MoS_2_. Reprinted with permission from Li et al. [78] Copyright 2018, Elsevier B.V.

In the TMDs hybridized with carbon, the carbon can be a supporting material, coated material, and intercalated material depending on the role and its length scale. Supporting carbon means the carbon that acts as a matrix for TMD materials. It is generally used for a carbon in a more or less macroscopic scale. Carbon coating generally means the carbon coated on the TMD surface. Intercalated carbon means the carbon that has been intercalated into layered TMDs in much smaller length scale (less than 1 nm). For example, glucose was converted to a polysaccharide through hydrothermal synthesis with abundant hydroxyl functional groups (Figure 13a) [139,140]. After converting it to the graphene foam as a carbon support, MoS_2_ was synthesized on this substrate to form a freestanding MoS_2_/graphene foam. The carbon coating of TMD nanoplatelets with other hybrid materials provides a large surface area and effectively inhibits of the restacking of the nanoplatelets [141,142,143]. Carbon coatings often form a graphene-like framework that improves the electrochemical performance of the cathode in ZIBs [78], MIBs [89,104], and AIBs [107]. Besides, the coated carbon enhances the ionic conductance of the TMD, which facilitates the diffusion of M^n+^ through the 3D foam structure (Figure 13b). Intercalated carbon has also been explored as an effective material for the interlayer expansion of TMDs (Figure 13c). Li et al. demonstrated the increase in MoS_2_ interlayer distance from 0.62 to 0.70 nm after carbon intercalation.

Over the past few decades, various methods have been proposed to control the morphology and structure of layered TMD hybrid materials. Hydrothermal/solvothermal synthesis methods are cost-effective and offer large product yields. The chemical vapor deposition method is a popular bottom-up method for developing high-quality layered TMD nanomaterials on many substrates. Exfoliation is a top-down method for preparing TMD nanosheets from bulk TMDs. As hybrids of layered TMDs with other materials show the merits of all the constituents, they are considered as potential candidates for application in energy storage devices [68,69,144]. Thus, the use of hybrid TMDs is an efficient approach for designing high-performance AMMIBs.

### 4.4. Phase Modification

The electronic structures of graphene and silicon are determined by the hybridization of their s and p orbitals, whereas the electronic structure of TMD layers is mainly affected by the d-orbital filling of the transition metal. The electron density of the d-orbitals in the transition metal affects the phase state of TMDs, and the electron filling of the d-orbitals causes phase modification in TMDs. Phase modification is often used to alter the electronic properties of a material. In general, phase modification in TMDs occurs incompletely, thereby resulting in the formation of mixed 1T and 2H phases.

**Figure 14:**
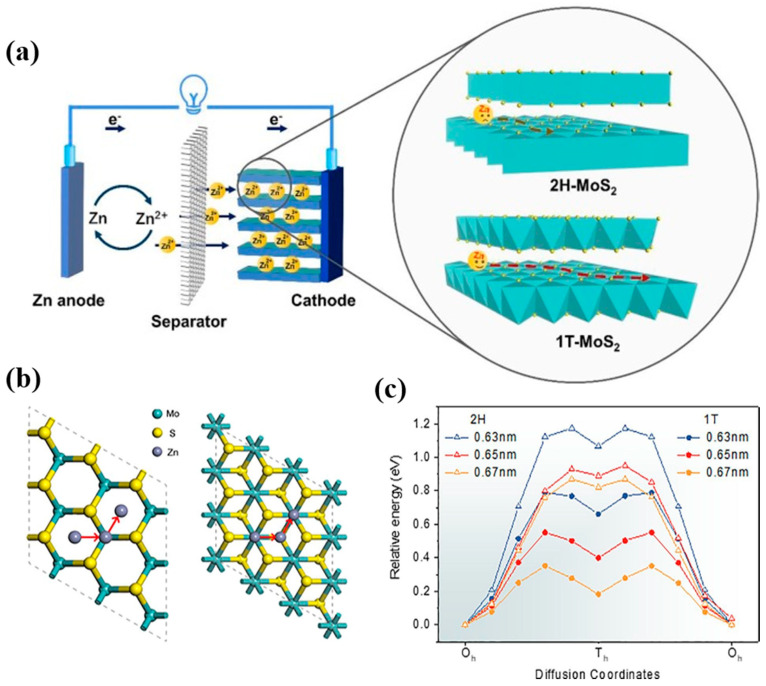
(**a**) Schematic of a rechargeable MoS_2_ cathode with different phases (1T- and 2H-MoS_2_). (**b**) The adsorption sites and diffusion pathway of Zn^2+^ (left:2H phase MoS_2_and right: 1T phase MoS_2_). (**c**) Calculation of Zn^2+^ diffusion energy barrier on the1T and 2H phases. Reprinted with permission from Liu et al. [75] Copyright 2020, Elsevier B.V.

As a representative TMD, MoS_2_ possesses different structural stages depending on the metal coordination geometry, properties, and stacking order. For instance, bulk MoS_2_ has two different phases: the 2H phase with a triangular prismatic coordinated geometry (bandgap of 1.3 eV) and the 1T phase with an octahedral coordination between Mo and S atoms (metallic properties), as shown in Figure 14a [63,145,146]. The ionic conductivity of the 1T MoS_2_ phase is much larger than that of the 2H MoS_2_ phase [147,148,149]. The different phases of MoS_2_ affect its electrochemical performance. For example, the phase engineering of MoS_2_ has been demonstrated to be an effective strategy for modifying its catalytic capacity [150,151,152,153]. Because of the different atomic and electronic structures of the hydrophobic 2H and hydrophilic 1T phases, the different concentrations of the two phases in MoS_2_ can significantly affect the adsorption sites and diffusion pathways for Zn^2+^ ions in rechargeable aqueous ZIBs (Figure 14b). Liu et al. proposed an effective method to produce MoS_2_ nanosheets with different phase contents through phase modification [75]. The MoS_2_ nanosheets with a high 1T phase content (~70%) showed better specific capacity and cycling performance in ZIBs than those with the 2H phase. In addition, as the distance between the interlayers increased, the diffusion energy barrier of Zn also decreased (Figure 14c).

## 5. Conclusion and Outlook

In this review, the potential of layered TMDs for application as AMMIB cathodes is discussed. TMDs have many advantages over other cathode materials: (i) it is facile to control the interlayer spacing; (ii) ion transport is fast benefited from layer structure; (iii) they are abundant in nature and environmentally friendly; (iv) they can be utilized easily for flexible battery applications owing to its mechanical flexibility; (v) they can be produced on a large-scale using exfoliation of bulk TMDs (top-down approach) and sol-gel synthesis (bottom-up approach). However, there are still challenges that should be overcome when using TMD as a cathode material in AMMIBs: (i) the electronic conductivities of most TMDs are not sufficiently high due to the nature of the semiconductor with a certain bandgap; (ii) a highly dispersed TMDs can be restacked during repeated charge/discharge processes due to interfacial instability among TMDs with few layers; (iii) the increase in surface area of TMDs tends to produce various byproducts (e.g., gel-like polymer) from electrolyte decomposition, leading to the irreversibility of the electrochemical reaction. Despite their sufficient interlayer spacing as compared to the size of M^n+^, TMDs suffer from ionic diffusion in aqueous environments because of the hydration effect, which increases the hydrodynamic size of M^n+^. 

Therefore, special efforts should be made to overcome these obstacles. Various strategies such as (1) intercalation modification, (2) defect modification, (3) hybridization, and (4) phase modification have been proposed to reduce the ion diffusion energy barrier, increase the interlayer spacing, improve the ion absorption, increase the electronic conductivity, and increase the hydrophilicity of TMD electrodes as discussed earlier. In addition, doping of appropriate materials in TMDs can increase the electronic conductivity especially for the wide bandgap TMDs. Moreover, as predicted from many recent theoretical studies using DFT calculations, the TMD heterostructure (e.g., MoS_2_/MoSe_2_, MoS_2_/WS_2_, etc.) can retain much higher theoretical capacity than single component TMDs. Toward this direction, a facile and reliable synthesis of TMD heterostructure is proposed as a promising opportunity.

Although great progress has been achieved in the development of TMD-based cathode materials for AMMIBs, extensive efforts are still required to design novel TMD cathode materials for future energy storage devices. It is still challenging to synthesize MX_2_ nanostructures with controllable size, interlayer distance, number of layers, phases, composition, and amounts of intercalated foreign species through facile, large-scale, and green synthesis methods. The correlation between the interlayer spacing of TMDs and the hydrodynamic size of ionic species is one of the basic factors to be considered for realizing high-performance AMMIBs. In addition, understanding the adsorption and diffusion mechanisms, origin of possible instability during intercalation/deintercalation, and the role of structural features in TMDs are all important aspects for designing novel TMD materials that can create new opportunities in AMMIBs.

To meet these expectations, a systematic and comprehensive study of characterization techniques will be beneficial. Various in situ analysis techniques will be helpful in monitoring the structural changes in MX_2_ during the intercalation of foreign species. Real-time measurements can provide important dynamic information regarding the origin of interlayer expansion. Density functional theory calculation is another useful method for understanding the role of interlayer engineering by quantitatively predicting the dependence of material properties on the interlayer spacing and intercalated species. The strategies and results summarized in this review will provide a guideline for realizing the potential applications of TMDs in future AMMIBs. 

## Figures and Tables

**Table 2:** Electrochemical performance of TMDs as zinc-ion batteries (ZIB) cathodes.

TMDs	Interlayer Spacing of Activated (Å)	Specific Capacity (mAh g^−1^)	Capacity Retention (%)	Cycle	Current Density (mA g^−1^)	Voltage Ranges (V)	Comments (Main Findings)	Ref.
MoS_2_	-	18.0	-	50	50	0.1–1.9	Study of zinc ion storage in pristine TMDs	[24]
WS_2_	-	22.0	-	50	50	0.1–1.9	Study of zinc ion storage in pristine TMDs	[24]
MoS_2_-O	9.5	232	-	20	100	0.2–1.4	Reduction in intercalation energy barrier by oxygen incorporation	[77]
E-MoS_2_	7.0	202.6	98.6	600	100	0.3–1.5	A novel structure of MoS_2_	[78]
1T-MoS_2_	6.8	-	98.4	400	100	0.25–1.25	Effect of different phase contents on the distinct performance	[75]
Defect-rich MoS_2_	6.86	88.6	87.8	1000	100	0.25–1.25	Development of defect rich MoS_2_ nanosheets	[79]
Tubular MoS_2_	6.5	146.2	74	800	200	0.25–1.25	A novel structure of MoS_2_	[80]
VS_2_	5.76	110.9	98	200	500	0.4–1.0	Storage mechanism of Zn/VS_2_	[81]
VS_2_@SS	5.8	198	80	2000	2000	0.4–1.0	Binder-free hierarchical VS_2_@SS electrode	[82]
VSe_2_	6.1	131.8	80.8	500	100	0.25–1.50	Zinc-ion transport behavior in VSe_2_ nanosheets	[83]
VS_4_	5.83	110	85	500	2500	0.2–1.6	Energy storage mechanism of VS_4_	[84]

**Table 3:** Electrochemical performance of TMD cathodes for magnesium-ion batteries (MIBs).

TMDs	Interlayer Spacing of Activated (Å)	Specific Capacity (mAh g^−1^)	Capacity Retention (%)	Cycle	Current Density (mA g^−1^)	Voltage Range (V)	Comments (Main Findings)	Ref.
Zigzag MoS_2_	-	170	-	-	-	-	Study of Mg adsorption sites with DFT calculations	[87]
G-WS_2_	0.7	161.5	95	50	20	0.5–3.0	A novel structure of G-MoS_2_ cathode and ultra-small Mg nanoparticle anode	[88]
MoS_2_/C	1.07	118.8	-	50	50	0–2.4	A novel structure of MoS_2_/C cathode and AZ31 Mg alloy anode	[89]
Bulk MoS_2_	-	81	68.7	10	20	0.2–2.2	Exfoliation of TMD into high-quality nanosheets	[90]
Bulk MoSe_2_	-	55	73.3	5	20	0.2–2.2	Exfoliation of TMD into high-quality nanosheets	[90]
TiS_2_	-	80	63.0	40	5	0.5–2.0	Kinetic study of Mg^2+^ migration in layered TMD	[91]
TiSe_2_	-	86	74.8	40	5	0.5–2.0	Kinetic study of Mg^2+^ migration in layered TMD	[91]
1T-TiSe_2_	-	108	-	40	-	0.25–1.8	Study of phase effect in TiSe_2_ on the battery performance	[92]
WSe_2_	6.8	239	91.9	100	50	0–2.5	A novel WSe_2_ structure	[93]

**Table 4:** Electrochemical performance of TMD cathodes for aluminum-ion batteries (AIBs).

TMDs	Interlayer Spacing of Activated (Å)	Specific Capacity (mAh g^−1^)	Capacity Retention (%)	Cycle	Current Density (mA g^−1^)	Voltage Range (V)	Comments (Main Findings)	Ref.
TiS_2_	-	70	-	50	5	0.2–1.3	Reversible insertion and extraction of Al in TiS_2_	[108]
MoS_2_ (E-MG)	1.0	87.6	-	120	20	0.01–2.0	A novel structure of MoS_2_ (E-MG)	[104]
MoS_2_	6.2	66.7	-	100	40	0.5–2.0	Phase transition mechanism during the charge-discharge process in MoS_2_	[105]
MoS_2_	6.3	30	-	-	100	0.01–2.5	Intercalation mechanism of Al^3+^ into MoS_2_	[106]
G-VS_2_	5.75	50	33.6	50	100	0.3–1.7	Intercalation mechanism of Al^3+^ into G-VS_2_	[107]
Mo_6_S_8_	-	70	-	50	-	0.1–1.2	Reversible intercalation and extraction of Al^3+^ in Mo_6_S_8_	[109]
